# Stellenwert der operativen Behandlung thorakolumbaler Wirbelkörperfrakturen für die Überlebenswahrscheinlichkeit alterstraumatologischer Patienten

**DOI:** 10.1007/s00113-020-00864-w

**Published:** 2020-09-15

**Authors:** Andreas Wiedl, Stefan Förch, Annabel Fenwick, Edgar Mayr

**Affiliations:** Abteilung für Unfallchirurgie, Orthopädie, Plastische und Handchirurgie, Universitätsklinikum Augsburg, Stenglinstraße 2, 86156 Augsburg, Deutschland

**Keywords:** Unfallchirurgisch-geriatrisches Co-Management, Mortalität, Kyphose, OF-Score, Wirbelkörperaugmentationen, Orthogeriatric co-management, Mortality, Kyphosis, OF-Score, Vertebral augmentation

## Abstract

**Hintergrund:**

Osteoporotische Wirbelkörperfrakturen sind eine häufige Verletzung alter Menschen, deren optimale Behandlung (konservativ oder operativ) diskutiert wird.

Die Literatur beschreibt nach Wirbelkörperaugmentationen geringere Mortalitäten als nach konservativer Therapie. Ob eine positive Korrelation des operativen Vorgehens mit dem Überleben nach oben genannten Verletzungen besteht, soll im eigenen alterstraumatologischen Patientenkollektiv überprüft werden.

**Methodik:**

Es erfolgte die Erfassung aller Patienten, die mit einer osteoporotischen Wirbelkörperfraktur vom 01.02.2014 bis 31.01.2015 auf einer alterstraumatologischen Station behandelt wurden. Im Rahmen eines 2‑Jahres-Follow-up wurden diese auf die assoziierte Sterblichkeit untersucht, wobei insbesondere der Einfluss der Therapie untersucht wurde.

**Ergebnisse:**

Insgesamt konnten 74 Patienten (Rücklauf 74 %) mit einem durchschnittlichen Alter von 83,2 Jahren eingeschlossen werden, davon wurden 40 konservativ und 34 operativ versorgt. Die gesamte Ein- und Zweijahresmortalität betrugen 29,7 % bzw. 35,1 %, nach operativer Versorgung 20,6 % bzw. 23,5 % und nach konservativer Therapie 37,5 % bzw. 45 % (*p* = 0,113 bzw. 0,086, Chi-Quadrat-Test). Die um Störfaktoren bereinigte „hazard ratio“ betrug 2,0 (95 %-KI: 0,686–6,100)

**Diskussion:**

Auch wenn möglicherweise wegen der eher geringen Fallzahl kein signifikantes Ergebnis nachgewiesen werden konnte, zeigen die Analysen eine Tendenz des verbesserten Überlebens nach operativem Vorgehen. Dies steht im Einklang mit internationalen Studien. Bestehende Untersuchungen lassen vermuten, dass die Reduktion der Kyphosierung durch die Operation einen wichtigen kausalen Zusammenhang darstellen könnte.

## Abstract (en)

**Background:**

Osteoporotic vertebral compression fractures (VCF) are a common injury among older patients. The optimal treatment option (operative or conservative) is still discussed. The literature describes *a* reduced mortality following operative augmentation of VCF compared to conservative treatment. We examined our orthogeriatric patient cohort to find out whether there is a positive correlation between surgical treatment of VCF and the survival rate.

**Methods:**

We performed an assessment of all patients with an osteoporotic spinal fracture who were treated on an orthogeriatric care unit due to VCF between 1 February 2014 and 31 January 2015. The treatment associated-mortality was examined in a 2-year follow-up, with a special focus on the influence of the treatment.

**Results:**

A total of 74 patients (74% follow-up) with an average age of 83.2 years were included, 40 having been treated conservatively and 34 surgically. Overall, the 1‑year and 2‑year mortalities were 29.7% and 35.1%, respectively. Surgical treatment was associated with 1‑year and 2‑year mortalities of 20.6% and 23.5% compared to 37.5% and 45%, respectively, after conservative treatment (*p* = 0.113 and 0.086, χ^2^-test). The adjusted hazard ratio was 2.0 (95% confidence interval, CI 0.686–6.100).

**Discussion:**

Although no statistically significant difference between the treatment groups could be detected (possibly due to the small sample size), the analysis showed a tendency towards an improved survival after surgical treatment. This is in accordance with international studies. Further investigations in the literature suggest that the reduction of kyphosis by surgery could represent an important causal association.

## Einleitung

Osteoporotische Wirbelkörperfrakturen werden in ihrer Häufigkeit und Relevanz für die Alterstraumatologie häufig unterschätzt. Ungefähr jede 5. Frau über 50 wird nach Schätzungen eine solche Verletzung zu Lebzeiten erleiden [21]. Die thorakolumbale Wirbelsäule betreffenden „Wirbelkörpereinbrüche“ weisen im Verlauf für Betroffene ein vergleichbares Sterberisiko zu proximalen Femurfrakturen auf [7]. Komplikationen nach initial stattgehabten Wirbelkörperfrakturen sind Anschlussfrakturen benachbarter Wirbelkörper [8] und weitere osteoporotische Frakturen des Skeletts [16]. Außerdem können die initialen Frakturen „sintern“ und zu einer zunehmenden Kyphosierung des betroffenen Abschnitts der thorakolumbalen Wirbelsäule führen [15]. Dieser Verlust der Integrität kann zu chronischen Schmerzen, Verlust der Aktivitäten des täglichen Lebens (eng. activities of daily living, ADL), psychischen Folgeerscheinungen und einer eingeschränkten Lungenfunktion führen [18, 20]. Zur Einteilung und zur Indikationsstellung wurde im deutschsprachigen Raum in den vergangenen Jahren vielerorts die OF-Klassifikation eingeführt. Neben der Bewertung der Frakturmorphologie werden in die Therapieentscheidung klinische Verlaufsparameter wie Schmerzniveau, Mobilisation, neurologische Ausfallserscheinungen, Knochendichte und Sinterung der Fraktur in Röntgenverlaufskontrollen miteinbezogen. Ein auf Basis dieser Parameter gebildeter Score empfiehlt das therapeutische Vorgehen [19]. Die konservative Therapie besteht i. Allg. in einer schnellstmöglichen Mobilisation unter adäquater Analgesie. Operative Stabilisierungen richten sich nach dem Grad der vorliegenden Deformität und umfassen Wirbelkörperaugmentierungen (Vertebroplastie, Kyphoplastie), ebenso wie dorsale Instrumentierungen und ventrale Stabilisationen. Insbesondere aufgrund der oben beschriebenen Komplikationen, die mit einer Kyphosierung des betroffenen Wirbelsäulenabschnitts einhergehen, drängt sich die Frage auf, inwiefern ein operatives, stabilisierendes Vorgehen einen positiven therapeutischen Einfluss für Patienten haben könnte. Zwei groß angelegte Studien, welche Daten des MediCare-Systems der USA auswerteten, geben Grund zur Annahme, dass das operative Vorgehen das Überleben der betroffenen Patienten deutlich verbessert [2, 4]. Auf einer alterstraumatologischen Station mit geriatrisch-unfallchirurgischem Komanagement wurden Patienten mit osteoporotischen Frakturen der thorakolumbalen Wirbelsäule erfasst. Primäres Ziel war die Ermittlung der Ein- und Zweijahresmortalitäten der Patienten in Anbetracht folgender Punkte:

- Vergleich der Therapie operativ zu konservativ,
- Frakturmorphologie nach der OF-Klassifikation,
- Einfluss von Störfaktoren (wie z. B. Komorbiditäten und Mobilität).

## Methodik

### Patientenerfassung

Alle Patienten, die sich auf der alterstraumatologischen Station vom 01.02.2014–31.01.2015 aufgrund einer gesicherten osteoporotischen Fraktur der thorakolumbalen Wirbelsäule in Behandlung befanden, wurden erfasst. Entsprechende Einwilligungen der Patienten oder der Betreuer lagen vor, ebenso ein positives Votum der Ethik-Kommission (7/11192). Im Rahmen des geriatrischen Assessment erfolgte die Erhebung des „Parker mobility score“ (PMS) [17], des Barthel-Index (BI) [13] sowie des „Charlson comorbidity index“ (CCI) [1].

Erfasst wurden die Frakturmorphologie anhand der OF-Klassifikation, der OF-Score sowie die erfolgte Therapie.

### Diagnostik und Therapie

Die Diagnosesicherung erfolgte mit konventionellem Röntgen und Computertomographie (CT). Eine Magnetresonanztomographie (MRT) wurde zur Identifikation eindeutig frischer Frakturen durchgeführt, sollten keine Kontraindikationen vorgelegen haben und keine Voraufnahmen der betreffenden Abschnitte der Wirbelsäule vorgelegen haben.

Die CT-morphologisch dargestellte Deformität des Wirbelkörpers wurde zunächst von dem diensthabenden Radiologen und Unfallchirurgen begutachtet. Anhand der OF-Klassifikation wurde diese dann im gemeinsamen Rapport der Abteilung eingeteilt. Leitliniengerecht erfolgte bei simpler Morphologie der Fraktur der konservative Therapieversuch mit einer frühzeitigen Mobilisation unter Analgesie [3]. Nach 3 bis 4 Tagen erfolgte eine Röntgenverlaufskontrolle im Stehen, um progrediente Sinterungen der Wirbelkörper zu identifizieren. Bei regelhaftem Therapieverlauf wurde die konservative Therapie fortgeführt.

Operative Verfahren wurden entsprechend der OF-Klassifikation indiziert, sollten eines oder mehrere der folgenden Merkmale vorgelegen haben:

- Fraktur nach OF-Klassifikation mit Frakturmorphologie ≥OF3 oder instabiler Fraktur,
- Patienten, die 5 Tage lang nicht ausreichend unter Analgesie mobilisierbar waren,
- zunehmender Kollaps des Wirbelkörpers in der Verlaufsröntgenkontrolle,
- neurologische Ausfallerscheinungen.

Die Indikation zur operativen Therapie wurde im gemeinsamen Konsens der Abteilung gefunden. Entsprechend dem OF-Score wurde auch bei höhergradiger Frakturmorphologie, aber guter Mobilität und ausreichender Analgesie keine Operation durchgeführt, vice versa bei Frakturen niederen Grades. Im Fall der Operation erfolgte bei einfacher Morphologie und stabiler Fraktur die Augmentation mittels Kyphoplastie. Bei höhergradigen oder instabilen Frakturen wurde eine dorsale Instrumentierung gewählt und ggf. um eine ventrale Stabilisation mittels Zementaugmentation ergänzt. Alle Patienten der alterstraumatologischen Station erhielten ein initiales geriatrisches Assessment. Im Gegensatz zu den konventionellen unfallchirurgischen Stationen wurden alle Patienten täglich von einem Geriater und Unfallchirurgen betreut (feste geriatrische Betreuung auf Station), erhielten täglich zweimalig Physio- und einmalig Ergotherapie und wurden aktiv-therapeutisch gepflegt. Für Betroffene und Angehörige stand, wie auch auf den konventionellen unfallchirurgischen Stationen, der soziale Beratungsdienst bei der Klärung der weiteren Versorgung oder der Rehabilitation zur Seite.

### Erfassung der Mortalitäten

Patienten und Angehörige wurden 2 Jahre nach dem stationären Aufenthalt über Fragebogen kontaktiert. Bei fehlendem Rücklauf erfolgte die telefonische Kontaktaufnahme mit den Patienten oder deren Angehörigen mit maximal 5 Versuchen. Das Versterben des Patienten und der Sterbemonat konnten so erfasst werden.

### Datenauswertung und Statistik

Die Auswertung wurde mittels des Statistik-Programms IBM SPSS Statistics Subscription (IBM Corp., Armonk, New York, USA) durchgeführt. Lineare Parameter wurden auf deren Streuungsbreite untersucht. Zur Vergleichbarkeit der Gruppen erfolgten T‑Tests für unabhängige Stichproben und der „Fisher’s exact test“ (FET), um signifikante Unterschiede in den Verteilungen des CCI, PMS, BI, Alters und Geschlechts aufzudecken. Bei den vorliegenden Sterbezahlen der Ein- und Zweijahresmortalitäten lagen Vierfeldertafeln vor. Die Signifikanztestung erfolgte mittels des Chi-Quadrat-Tests (CQT) und des FET mit 5 %-Signifikanzniveaus. Da die genauen Sterbezeitpunkte vorlagen, konnten Kaplan-Meier-Kurven erstellt werden, diese wurden mithilfe des „log-rank test“ (LRT) auf signifikante Unterschiede überprüft. Zur Bestimmung des relativen Sterberisikos, der „hazard ratio“ (HR) zwischen den Subgruppen, konnte die Cox-Regression-Analyse genutzt werden.

## Ergebnisse

Im Beobachtungszeitraum wurden insgesamt 100 Patienten bei Verletzungen der thorakolumbalen Wirbelsäule auf der alterstraumatologischen Station behandelt. Von 74 konnten Zweijahresverlaufsdaten erhoben werden, was einem Rücklauf von 74 % entspricht. Das durchschnittliche Alter der Patienten betrug 83,24 ± 6,15 Jahre. Die Geschlechterverteilung betrug ♂:♀ = 3:7, bei 22 behandelten Männern und 52 behandelten Frauen. Es wurden 34 (45,9 %) Patienten operativ und 40 (54,1 %) konservativ behandelt. Wie Tab. 1 zu entnehmen ist, bestanden zwischen den Gruppen keine signifikanten Verteilungsunterschiede bezüglich des Alters, Geschlechts, PMS, CCI und BI. Dennoch ließ sich erkennen, dass sich im konservativen Kollektiv immobilere (PMS) und mehr männliche Patienten befanden, wohingegen im operativen etwas kränkere Patienten (CCI) behandelt wurden. Es wurden 21 Kyphoplastien, 9 singuläre dorsale Stabilisationen und 4 dorsale Stabilisationen mit Zementaugmentation der betroffenen Wirbelkörper durchgeführt. Die einzelnen Verfahren werden in Tab. 2 angegeben, hier wird zudem die jeweilige konservative Schmerztherapie nach WHO beschrieben. In der operativen Gruppe befanden sich Patienten mit signifikant höherem OF-Score und signifikant höhergradiger OF-Morphologie (Tab. 1). Die Zahl der Patienten in Abhängigkeit von der Frakturmorphologie zeigt Tab. 4. Ungefähr die Hälfte der Patienten wies somit die Frakturmorphologie OF 2 auf. Vier konservativ geführte Patienten wiesen die Morphologie OF 4 auf, obwohl nach oben genannten Kriterien die Operation prinzipiell indiziert gewesen wäre. Zwei dieser Patienten wurden aufgrund multipler Vorerkrankungen, einer im Sinne einer Individualentscheidung und ein weiterer beim Versterben während des Aufenthalts nicht operiert.

**Table 1:** 

		Operative Gruppe	Konservative Gruppe	Gesamt	*p*-Werte
Durchschnittliches Alter		82,79 (80,80–84,53) Jahre	83,63 (82,74–84,53) Jahre	83,24 (81,82–84,67) Jahre	0,566
Geschlecht	Männlich	7	15	22	0,133
	Weiblich	27	25	52	
Parker mobility score		5,91 (5,09–6,73)	4,83(−3,91–5,75)	5,35 (4,73–5,97)	0,081
Barthel-Index		29,70 (24,82–34,57)	27,86 (22,39–33,32)	28,14 (24,55–31,74)	0,549
Charlson comorbidity index		3,24 (2,25–4,23)	2,8 (2,07–3,53)	2,99 (2,41–3,57)	0,212
OF-Score		7,32 (6,68–7,96)	2,28 (1,74–2,81)	4,60 (3,88–5,31)	0,000
OF-Morphologie		3,15 (2,71–3,59)	2,25 (2,00–2,50)	2,66 (2,40–2,92)	0,000

Angabe der *p*-Werte nach Testung der Gruppen operativ und konservativ auf signifikante Unterschiede

**Table 2:** 

	Verfahren und WHO Stufen	Anzahl	Operationsdauer in Minuten (Median, Minimal- und Maximalwerte)	Zahl der Operateure (*n*)
Operative Verfahren (*n* = 34)	Kyphoplastie	21	34; 12–79	14
	Minimalinvasiver Fixateur interne	9	75; 31–122	6
	Minimalinvasiver Fixateur interne + Kyphoplastie	4	145; 119–171	3
Konservative Therapie (*n* = 40)	WHO-Stufe 1	0	–	–
	WHO-Stufe 2	6	–	–
	WHO-Stufe 3	34	–	–

### Ein- und Zweijahresmortalitäten

Die Vierfeldertafeln jeweils für die Ein- und die Zweijahresmortalitäten zeigt Tab. 3. Die gesamte Einjahresmortalität betrug 29,7 % (22/74) und die Zweijahresmortalität 35,1 % (26/74).

**Table 3:** 

	Alle Patienten	Operative Gruppe	Konservative Gruppe
Anzahl	74	34	40
Einjahresmortalität	29,7 % (22/74)	20,6 % (7/34)	37,5 % (15/40)
Zweijahresmortalität	35,1 % (26/74)	23,5 % (8/34)	45,0 % (18/40)

Die Einjahresmortalität ergab für die konservative Gruppe 37,5 % (15/40) und für die operative Gruppe 20,6 % (7/34), bei einem *p*-Wert von 0,113 im CQT.

Die Zweijahresmortalität betrug für die konservative Gruppe 45 % (18/40) und für die operative Gruppe 23,5 % (8/34). Hier zeigte sich im FET ein *p*-Wert von 0,086, somit lag ein knapp nichtsignifikantes Ergebnis bei deutlicher Tendenz zur geringeren Sterblichkeit der operativen Gruppe vor.

**Figure 1:**
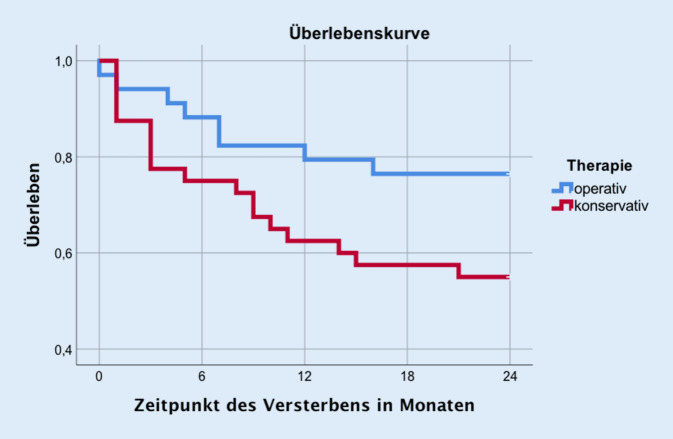

**Figure 2:**
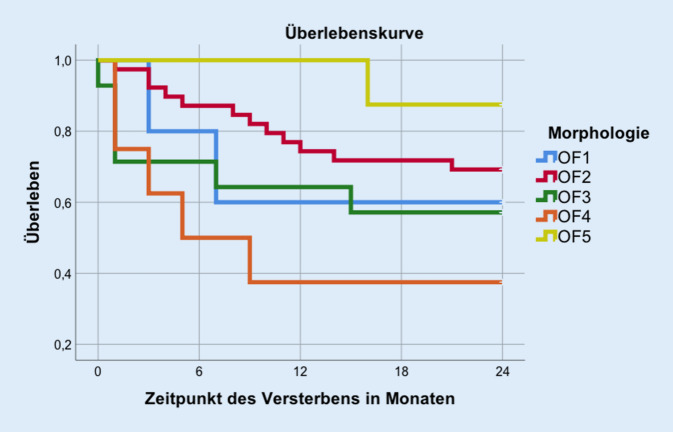

Den Sterblichkeitsverlauf der Gruppen untereinander verdeutlicht Abb. 1, im Vergleich der Kurven zeigte sich ein *p*-Wert von 0,06 im LRT. Die rohe HR im Vergleich konservative vs. operative Therapie betrug 2,2 (95 %-KI: 0,94–4,98). Somit war das relative Sterberisiko der Patienten, die nicht operiert wurden, um den Faktor 2,2 erhöht. In Tab. 4 werden die Zweijahresmortalitäten für die unterschiedlichen Frakturmorphologien dargestellt. Hier findet sich eine niedrigere Mortalität der operativen Gruppe, unabhängig vom vorliegenden Frakturtyp. Patienten der Gruppe OF 5 verzeichneten die geringste Sterblichkeit (Abb. 2).

**Table 4:** 

	Alle Patienten		Operative Gruppe		Konservative Gruppe	
	*n*	2JM (%)	*n*	2JM (%)	*n*	2JM (%)
OF 1	5	40	1	100,0	4	25,0
OF 2	39	30,8	13	15,4	26	38,5
OF 3	14	42,9	8	25,0	6	66,7
OF 4	8	62,5	4	50,0	4	75,0
OF 5	8	12,5	8	12,5	0	0,0

Zahl der Patienten mit der jeweils vorliegenden Frakturform nach OF und der assoziierten Zweijahresmortalitäten (*2JM*) für alle Patienten und therapiespezifisch

### Multivariate Analyse mittels Cox-Regression

In Tab. 5 erfolgt die Darstellung der multivariaten Analyse mittels Cox-Regression. Miteinbezogen sind die Faktoren Therapie, Frakturmorphologie, Alter, Geschlecht, PMS, CCI und BI. Die bereinigte HR der Gruppe konservativ vs. operativ betrug 2,0 (95 %-KI: 0,686–6,100). Auch wenn hier weiterhin kein statistisch signifikanter Zusammenhang nachgewiesen wurde, besteht doch auch weiterhin eine deutliche Tendenz. Konservativ geführte Patienten verstarben mit einer 2‑fach höheren Wahrscheinlichkeit als operierte Patienten. Außerdem lässt sich erkennen, dass Verzerrungseffekte durch „confounder“ praktisch nicht vorhanden sind (die bereinigte und die rohe HR sind unwesentlich unterschiedlich).

**Table 5:** 

	Sig.	HR	95,0 %-KI der HR	
			Untere	Obere
Konservativ vs. operativ	0,200	2,045	0,686	6,100
Geschlecht	0,433	0,699	0,285	1,712
Alter	0,125	1,066	0,982	1,158
Frakturmorphologie	0,866	1,039	0,668	1,616
Barthel-Index	0,474	0,631	0,179	2,228
Charlson comorbidity index	0,022	2,225	1,124	4,404
Parker mobility score	0,134	0,633	0,348	1,151

Berechnung des relativen Sterberisikos (HR) der konservativen zur operativen Gruppe unter Ausschaltung der beschriebenen Störfaktoren. Die HR für die anderen Faktoren beschreibt folgende Relationen: Alter x+1:x, Geschlecht weiblich zu männlich, Frakturmorphologie nach OF x+1:x, BI 70–100:35–65:0–30, CCI 0–1:2–3:≥4, PMS 7–9:4–6:0–3

Ein statistischer Zusammenhang zwischen Frakturmorphologie und Mortalität konnte nicht nachgewiesen werden. Es ließ sich für steigendes Alter ebenfalls kein signifikanter Effekt nachweisen. Für Frauen bestand ein nichtsignifikanter Überlebensvorteil mit einer HR von 0,70 (95 %-KI: 0,285–1,712). Bessere Funktion und Mobilität im Sinne des BI und PMS erwiesen sich als prognostisch günstig bei einer HR von 0,63 (95 %-KI: 0,179–2,228) bzw. 0,63 (95 %-KI: 0,348–1,151), ohne *p*-Werte <0,05 vorzuweisen. Als einzige unabhängige Größe erwies sich die Zahl der Komorbiditäten, gemessen am CCI. Eine Zunahme der Komorbiditäten bedeutete eine stufenweise HR von 2,2 (95 %-KI: 1,124–4,404).

## Diskussion

Die aktuelle Studie untersuchte die mit osteoporotischen Wirbelkörperfrakturen assoziierte Mortalität konservativ und operativ behandelter Patienten über 2 Jahre. Dies geschah mit dem Ziel, den Einfluss der Therapie auf die Sterblichkeit zu ermitteln. Störfaktoren wie Alter, Geschlecht, ADL, Mobilität und Komorbiditäten konnten berücksichtigt und damit deren Einfluss und Verzerrungspotenzial auf das Ergebnis bestimmt werden. Mit im Follow-up erfassten 74 Patienten mit osteoporotischen Frakturen der thorakolumbalen Wirbelsäule handelt es sich im Vergleich zur Literatur eher um ein kleines Kollektiv. Vergleichsstudien betrachten deutlich größere Patientengruppen, teilweise weit über 1000 [2, 4, 7]. Dies sollte bei der statistischen Auswertung der Daten bedacht werden, da signifikante Effekte möglicherweise aufgrund der geringen Fallzahl verschleiert wurden.

Die Ein- und Zweijahresmortalität nahmen im Patientenkollektiv vergleichbare Werte zur Literatur an; von Johnell et al. werden hier Werte von 28 % bzw. 40 % beschrieben [7]. Analog dazu werden für proximale Femurfrakturen Einjahresmortalitäten von 23,4–29 % [5, 6, 12] und Zweijahresmortalitäten von 31–36 % [5, 7] angegeben.

Der therapiespezifische Vergleich der Mortalitäten bestätigte keinen signifikanten Überlebensvorteil der operierten Patientenklientel gegenüber der konservativ geführten, auch wenn die rohe bzw. die bereinigte HR 2,2 bzw. 2,0 betrugen. Damit war das Sterberisiko der nichtoperierten Patienten im Vergleich um den Faktor 2 erhöht. Störfaktoren, wie Mobilität, Funktion, Multimorbidität, Alter, Frakturmorphologie und Geschlecht, hatten keinen deutlichen Einfluss auf die HR. Im Vergleich des Einflusses der Frakturmorphologien zeigten sich keine signifikanten Überlebensvorteile einer minderschweren Fraktur. Interessant ist die äußerst niedrige Mortalität der Gruppe OF 5. Hier wurden v. a. Patienten mit Hyperextensionsverletzungen behandelt, welche mit keiner Wirbelkörperhöhenminderung einhergingen.

Chen et al. und Edidin et al. werteten Daten aus dem Medicare-System der USA aus; beide verglichen die Sterblichkeiten konservativ behandelter Patienten nach Wirbelkörperfrakturen mit denen, welche eine Vertebroplastie oder Kyphoplastie erhielten [2, 4]. Chen et al. untersuchten dabei ein Kollektiv von ca. 70.000. In diesem betrug das Überleben nach 3 Jahren für die konservative Gruppe 42,3 %, für die Vertebroplastiegruppe 49,7 % und für die Kyphoplastiegruppe 59,9 %. Edidin et al. konnten bei über 1 Mio. Patienten ein signifikant erhöhtes Sterberisiko nach konservativ behandelten Wirbelkörperfrakturen nachweisen. Dieses war hier gegenüber stattgehabter Vertebroplastie um 25 % und gegenüber stattgehabter Kyphoplastie um 55 % erhöht. Somit konnte in beiden Studien nachgewiesen werden, dass die Kyphoplastie, welche einer Kyphosierung des Wirbelkörpers entgegenwirkt, einen noch größeren Effekt auf das Überleben hat. Dennoch gibt es aber auch Studien wie von McCullough et al., welche nach Regressionsanalyse keinen signifikanten Effekt der Wirbelkörperaugmentation auf das Überleben nach einem Jahr fanden (Die Dreißigtagemortalität war jedoch immer noch signifikant niedriger als in der nichtoperierten Klientel. Außerdem lag eine im Vergleich relativ kleine operative Klientel von ca. 10.000 gegenüber einer konservativer Klientel von ca. 115.000 vor) [14]. Auch Lavelle et al. konnten analog nach multivariater Analyse keinen Unterschied im Überleben nachweisen, hier lag allerdings auch eine geringere Patientenzahl von 184 vor [11].

Oben genannte Studien legen einen relevanten Einfluss der Operation auf das Überleben nach Wirbelkörperfrakturen nahe. Interessanterweise schien die Aufrichtung des Wirbelkörpers in der Literatur nachgewiesenermaßen einen stärkeren Einfluss auf das Überleben zu haben als die reine Zementaugmentation. Möglicherweise muss dem Einfluss des Wirbelkörperkollapses und der Kyphosierung mehr Aufmerksamkeit zukommen. Verstärkt wird diese Vermutung durch die oben beschriebene geringere Mortalität der Gruppe OF 5 im eigenen Kollektiv.

Kado et al. wiesen unabhängig von einer vorbestehenden Osteoporose oder Wirbelkörperfraktur eine Korrelation des Grades der bestehenden Kyphose bei Frauen mit der Zunahme des Sterberisikos nach [9]. Ursächlich könnte eine durch die Kyphose bedingte Einschränkung der Lungenfunktion sein. So konnten Krege et al. eine Abnahme der Vitalkapazität in negativer Korrelation zu Zahl und Schweregrad vorliegender Wirbelkörpereinbrüche nachweisen [10]. Des Weiteren ist eine Augmentierung effektiver in der Reduktion des Schmerzniveaus als die konservative Therapie [22]. Ein Einfluss einer besseren Analgesie auf die Mobilisation und Funktion sowie der seltenere Gebrauch von Analgetika könnten einen weiteren kausalen Zusammenhang darstellen.

### Limitationen

Bei der durchgeführten Studie handelte es sich um eine prospektive Beobachtungsstudie. Es wurden alle Patienten der alterstraumatologischen Station erfasst. Dieser Umstand muss als möglicher Selektionsbias berücksichtigt werden. Die Einteilung der Therapiegruppen erfolgte nach einem vorgegebenen Algorithmus. Keine der Variablen Geschlecht, PMS, CCI, BI oder Alter zeigte einen signifikanten Verteilungsunterschied zwischen den Gruppen. Dennoch ließ sich erkennen, dass sich in der konservativen Gruppe mehr Männer und immobilere Patienten befanden. Um Verzerrungseffekte zu minimieren, wurden alle Störfaktoren in der Analyse berücksichtigt. Dennoch könnten Kovariablen, welche einen Einfluss auf die Verteilung innerhalb der Gruppen genommen haben, unerkannt geblieben sein. Eine sichere Elimination dieses Effekts liegt deshalb nicht vor. Die oben genannten Störfaktoren PMS, CCI und BI konnten nicht lückenlos erfasst werden, was deren gemeinsame Berücksichtigung in der multivariaten Analyse erschwert. Insgesamt wurden 4 Patienten, die eine OF-Morphologie 4 aufwiesen, aus verschiedenen genannten Gründen nicht operiert, obwohl bei diesen nach dem Therapiealgorithmus eine Operation prinzipiell indiziert gewesen wäre. Dies könnte die Sterblichkeit der konservativen Gruppe im Vergleich zur operativen Gruppe erhöht haben. Die eher geringe Fallzahl von insgesamt 74 Patienten bedingt in der statistischen Analyse möglicherweise eine Verschleierung eines statistisch signifikanten Effekts. Für künftige Analysen wäre deshalb ein größeres Patientenkollektiv, dessen Stärke ggf. durch eine Power-Analyse evaluiert wurde, wünschenswert.

## Fazit für die Praxis

- Die vorliegende Studie zeigt keinen signifikanten Unterschied in der Mortalität für Patienten nach operativer Behandlung osteoporotischer Wirbelkörperfrakturen im Vergleich zur konservativen Therapie.
- Die Ergebnisse zeigen jedoch eine tendenziell höhere Mortalität nach konservativer Behandlung; eine Bedeutung könnte die zunehmende Kyphose nach konservativer Therapie haben.
